# Harnessing inter-kingdom metabolic disparities at the human-fungal interface for novel therapeutic approaches

**DOI:** 10.3389/fmolb.2024.1386598

**Published:** 2024-04-24

**Authors:** Claudio Costantini, Marilena Pariano, Matteo Puccetti, Stefano Giovagnoli, Gioena Pampalone, Mirco Dindo, Barbara Cellini, Luigina Romani

**Affiliations:** ^1^ Department of Medicine and Surgery, University of Perugia, Perugia, Italy; ^2^ Department of Pharmaceutical Sciences, University of Perugia, Perugia, Italy

**Keywords:** fungal infections, mycobiota, metabolic network, inflammation, functional biochemistry

## Abstract

Humans interact with a multitude of microorganisms in various ecological relationships, ranging from commensalism to pathogenicity. The same applies to fungi, long recognized for their pathogenic roles in infection–such as in invasive fungal diseases caused, among others, by *Aspergillus fumigatus* and *Candida* spp.–and, more recently, for their beneficial activities as an integral part of the microbiota. Indeed, alterations in the fungal component of the microbiota, or mycobiota, have been associated with inflammatory, infectious and metabolic diseases, and cancer. Whether acting as opportunistic pathogens or symbiotic commensals, fungi possess a complex enzymatic repertoire that intertwines with that of the host. In this metabolic cross-talk, fungal enzymes may be unique, thus providing novel metabolic opportunities to the host, or, conversely, produce toxic metabolites. Indeed, administration of fungal probiotics and fungi-derived products may be beneficial in inflammatory and infectious diseases, but fungi may also produce a plethora of toxic secondary metabolites, collectively known as mycotoxins. Fungal enzymes may also be homologues to human enzymes, but nevertheless embedded in fungal-specific metabolic networks, determined by all the interconnected enzymes and molecules, quantitatively and qualitatively specific to the network, such that the activity and metabolic effects of each enzyme remain unique to fungi. In this Opinion, we explore the concept that targeting this fungal metabolic unicity, either in opportunistic pathogens or commensals, may be exploited to develop novel therapeutic strategies. In doing so, we present our recent experience in different pathological settings that ultimately converge on relevant trans-kingdom metabolic differences.

## 1 Introduction

The human genome was predicted to code for 2,709 enzymes assigned to 135 predicted metabolic pathways (Romero et al., 2005). Current estimates expands the list of all enzymes in *Homo sapiens* to 3,159 (humancyc.org; accessed 18 September 2023). This enzymatic repertoire delineates a metabolic map that defines the life cycle of macromolecules entering into, or metabolized within, its borders. It is becoming increasingly clear that the human metabolic map is not isolated, but share pathways–either in a transient or a stable manner–with the metabolic maps of each organism it encounters. From a molecular point of view, these connections among maps result in the integration of metabolites and metabolic activities among organisms, which may translate into different forms of association. Indeed, from an ecological point of view, the interaction may be beneficial, neutral or detrimental for the organisms that have come into contact. Translating these concepts into the human realm, the interaction with the commensal microorganisms that colonize the surfaces exposed to the external environment, or microbiota, is an example of beneficial interaction (Costantini, 2022). For example, the fermentation of nondigestible polysaccharides by gut bacteria results in the production of short-chain fatty acids (SCFAs), such as butyrate, a major energy source for colonocytes and a promoter of immune tolerance at the mucosal barrier. Similarly, microbial pathways of tryptophan (Trp) metabolism result in the production of indolic compounds, such as indole-3-aldehyde (3-IAld), which promotes epithelial barrier integrity in a variety of pathological settings. Conversely, the host’s local interaction with a potential pathogen may be detrimental, via the production of toxic metabolites or destabilization of host’s own homeostatic pathways. When focusing on the enzymatic processes shaping the metabolic pathways of individual organisms, the result of interactions between organisms is ultimately manifested in the flow of metabolites among these pathways and the distinct impacts these metabolites have within each one.

Fungi are eukaryotic organisms along with plants and animals with unique morphological, physiological, and nutritional traits (Nagy et al., 2017), which contribute to their profound impact on human hosts (Niego et al., 2023). In terms of human health, interest in fungi has grown exponentially in two opposite directions: first of all, the recognition of the major impact of fungal diseases that has prompted the World Health Organization to launch a list of priority fungal pathogens (Table 1 and (Rodrigues and Nosanchuk, 2023)); as another consequence, the presence of commensal communities of fungi that colonize several sites of the human body (Cui et al., 2013), with implications in human health and disease. From a genomic point of view, fungal genomes are generally small compared to the other eukaryotic plants and animals (Tunlid and Talbot, 2002), and non-uniform across taxonomically defined fungi (Merenyi et al., 2023), in line with the notion that fungal genomes are highly diverse (Mohanta and Bae, 2015). The concurrent metabolic diversity (Steindorff et al., 2015) translates in an ability of fungi to colonize most ecological niches and adapt to changing environmental conditions (Wisecaver et al., 2014), including host niches and their associated commensal bacteria (Brock, 2009). For instance, the host immune status and the microbiome are critical factors in the cross-talk between *Candida albicans* and its host. Indeed, the commensal nature of *C. albicans* may turn into a pathogenic one when the mucosal homeostasis is compromised, as observed in the course of antibiotic and/or chemotherapeutic treatments, which alter the composition and function of the bacterial communities, the integrity of the epithelial lining, and the interaction with the host immune system (Brock, 2009; Romani, 2011). Similarly, the environmental fungus *Aspergillus fumigatus* may cause respiratory infections in immunocompromised individuals, a process that requires not only an intrinsic metabolic adaptation to the host environment (van de Veerdonk et al., 2017) but also a rewiring process of the metabolism in the lung microbiome to be favorable for its growth (Mirhakkak et al., 2023).

**TABLE 1:** WHO fungal priority pathogens list (adapted from Rodrigues and Nosanchuk, 2023).

Critical priority group	*Cryptococcus neoformans*
	*Candida auris*
	*Aspergillus fumigatus*
	*Candida albicans*
High Priority Group	*Nakaseomyces glabrata* (*Candida glabrata*)
	*Histoplasma* spp.
	Eumycetoma causative agents
	Mucorales
	*Fusarium* spp.
	*Candida tropicalis*
	*Candida parapsilosis*
Medium Priority Group	*Scedosporium* spp.
	*Lomentospora prolificans*
	*Coccidioides* spp.
	*Pichia kudriavzeveii* (*Candida krusei*)
	*Criptococcus gattii*
	*Talaromyces marneffei*
	*Pneumocystis jirovecii*
	*Paracoccidioides* spp.

Drawing from these foundational principles, we aim to share our recent insights into exploring fungal metabolism at the interface with humans, with the goal of uncovering innovative therapeutic avenues. Initially, we will delve into the complex and context-dependent metabolism of the amino acid Trp. Here, the interplay of shared or distinct enzymatic activities and metabolic byproducts among the host, commensal microbes, and fungi significantly influences the outcome of their interactions. Subsequently, we will examine two specific pathological conditions to underscore how this context-driven approach, integrating fungal and host metabolic maps, can be leveraged to modulate host-fungal interactions in both health and disease settings.

## 2 A case for tryptophan metabolism

As previously mentioned, *A. fumigatus* is an environmental fungus and continuously enter into contact with humans via inhalation of fungal spores. While the immunocompetent host can efficiently clear the spores, a defective immune response may impair the ability of the host to eradicate the fungus, paving the way to colonization and infection (van de Veerdonk et al., 2017). Immunocompromised individuals, or patients with pre-existing conditions, may experience invasive aspergillosis, with a very high mortality rate, or other forms of colonization and/or infection, such as chronic pulmonary aspergillosis or allergic bronchopulmonary aspergillosis (van de Veerdonk et al., 2017). The complex interaction between the host and the fungus involves multiple levels of metabolic adaptation. Indeed, on the one hand, the fungus must adapt to the host environmental conditions by undergoing metabolic and stress responses (van de Veerdonk et al., 2017), while, on the other hand, the host needs to mount a balanced immune response to prevent both fungal colonization and collateral tissue damage (Romani, 2011). In this resistance vs. tolerance paradigm, the metabolism of the amino acid tryptophan (Trp) is emerging a central component. Trp metabolism comes with a series of interesting characteristics. From a structural point of view, Trp contains an indole, a bicyclic ring formed by benzene and pyrrole groups, which define a vast group of indole-containing bioactive molecules originating by the enzymatic metabolism of Trp. Of note, Trp might work as a substrate for various enzymes whose expression may be shared or differ between the host and its commensal microbes. In humans, the major amount of Trp is metabolized along the kynurenine pathway. The rate-limiting step is represented by the first reaction converting Trp to N-formylkynurenine and is mediated by three different enzymes that act as dioxygenases (Platten et al., 2019), namely, tryptophan 2,3-dioxygenase (TDO), indoleamine 2,3-dioxygenase (IDO)1 and IDO2. The three enzymes differ under several respects, including size, shape, location and enzymatic activity (Dolsak et al., 2021). Among them, indoleamine 2,3-dioxygenase (IDO)1–whose expression can be induced by inflammatory stimuli–plays a major role in the regulation of the immune response. Indeed, by causing Trp deprivation and inducing the production of kynurenines, IDO1 restrains the inflammatory response and promotes tolerance by inducing the generation of regulatory T cells. From an evolutionary perspective, TDO is found in almost all eukaryotes and bacteria, but not in fungi (Yuasa and Ball, 2015), while IDO genes can be found in both fungi (Yuasa and Ball, 2011) and bacteria (Yuasa et al., 2011).

Microbes are also able to metabolize Trp along different pathways, mainly involving the activity of tryptophanase, tryptophan decarboxylase, aromatic amino acid aminotransferases, and tryptophan monooxigenase, generating indole and a number of indole derivatives (Liu et al., 2020). For instance, indole generated by the activity of tryptophanase can be converted in indoxyl and indoxyl sulfate, a well-known uremic toxin (Berg et al., 2022). The activity of aromatic amino acid aminotransferases results in the production of indole-3-pyruvic acid, which in turn works as a substrate for the generation of other indolic compounds, such as indole-3-lactic acid and indole-3-propionic acid, both contributing to the maintenance of intestinal mucosa homeostasis. Within Lactobacilli, *L. reuteri* was found to convert Trp to indole-3-aldehyde (3-IAld) via the aromatic amino acid aminotransferase (Zelante et al., 2013). Mice exposed to a Trp-enriched diet expand *L. reuteri* in the gut that produce 3-IAld; this promotes AhR-dependent transcription of the IL-22–encoding gene by host innate lymphoid cells and thus prevents microbial infections and local inflammation (Zelante et al., 2013). 3-IAld, like indoles, moonlights as a metabolite and signaling molecule and is increasingly being associated with the regulation of wide-ranging physiological processes (Puccetti et al., 2023). In murine models, endogenous or administered 3-IAld was associated with resistance to infections, ionizing radiation, inflammaging, and gut, cerebral, and liver inflammation via a plethora of mechanisms, including the production of IL-22 and type I interferons, the modulation of IL-10 and IL-10R expression, NF-kB and TLR7 activity, the promotion of epithelial barrier function, and cross-talk with enteric neurons and the microbiota (Zelante et al., 2021a; Puccetti et al., 2023). Because an alteration in the microbial composition may change the levels of 3-IAld in human diseases (Puccetti et al., 2023), this observation suggests that replacement therapy with 3-IAld may be of benefit in human inflammatory diseases.

It is worth noting that humans can also metabolize Trp along the serotonin pathway. The rate-limiting step is catalyzed by the activity of Tryptophan Hydroxylase (Tph)1 and Tph2, with different size and kinetic properties as well as non-overlapping expression patterns (McKinney et al., 2005), which allows to create two independent systems for serotonin synthesis. In particular, while Tph2 is expressed in the brain and contributes to the central levels of serotonin, Tph1 is expressed by enterochromaffin cells in the intestine and is responsible for its peripheral levels (Walther et al., 2003). Long recognized for its role in the regulation of neuronal activities, serotonin/5-hydroxytryptamine (5-HT) may have an immune regulatory role in aspergillosis. Specifically, our group showed that 5-HT promoted pathogen clearance and immune homeostasis in infection by balancing between the host IDO1/kynurenine pathway and the microbial indole/AhR pathway (Renga et al., 2023). Interestingly, some bacteria, including strains present in the human gut, were also shown to produce serotonin, at least *in vitro* (Koopman et al., 2021).

Taken together, the combined activation of host and microbial Trp metabolic pathways converge towards immune homeostasis at mucosal surfaces, including an optimal immune response to fungi. An additional layer of complexity has come by the recent appreciation that *A*. *fumigatus* is not excluded from this common table. Indeed, as previously mentioned, tryptophan-degrading enzymes are also present in fungi (Yuasa and Ball, 2012; Koper et al., 2022). Specifically, *A*. *fumigatus* contains three indoleamine 2,3-dioxygenase (*ido*) genes, that metabolize Trp to kynurenines, and two aromatic aminotransferases (*aro*) genes, that convert Trp to indolepyruvate via the Ehrlich pathway (Choera et al., 2017). This enzymatic repertoire allows *A*. *fumigatus* to metabolize Trp in a context-dependent manner when adapting to the host niche (Zelante et al., 2021b). Specifically, environment-induced switching between the two Trp metabolic pathways dramatically influences the host-fungal interaction, resulting in either immune tolerance or inflammatory pathology (Zelante et al., 2021b). Therefore, integration of environmental cues rewires the fungal metabolic map to alter the flux of metabolites into the host metabolic map with opposite functional effects, paving the way to therapeutic interventions to prevent fungal virulence and promote immune tolerance. These intricate interconnections between host and fungal metabolic maps may also apply to the interaction between the host and the commensal *C. albicans*. We have already shown that IDO1 inhibition greatly exacerbated mucosal *C. albicans* infection and associated inflammatory pathology as a result of deregulated innate and adaptive/regulatory immune responses, an effect in line with the induced IDO1 activity at sites of infection (Bozza et al., 2005). However, a role for Trp catabolism was also demonstrated in a fungus-autonomous fashion in that its blockade *in vitro* promoted yeast-to-hyphal transition (Bozza et al., 2005). More recently, deletion of the ido gene encoding the enzymes of the kynurenine pathway greatly increased the virulence of the fungus *in vivo*, particularly if associated with host IDO1 deficiency (Romani L, unpublished observation), a finding further pointing to the central role of metabolic maps centered around the amino acid Trp at the host–fungus interface.

## 3 The case of sphingosine-1-phosphate lyase

Cystic Fibrosis (CF) is the most common autosomal recessive disease in the Caucasian population caused by mutations in the gene encoding for the Cystic Fibrosis Transmembrane conductance Regulator (CFTR), an ion channel that regulates the ionic content of luminal fluid. CF is a multi-organ disease although the pulmonary decline represents the major cause of morbidity and mortality (Bell et al., 2020). The defective CFTR function results in the formation of a viscous mucous layer that, on the one hand, impairs mucociliary clearance thus favoring microbial persistence and pathogen infection, and, on the other hand, damages epithelial cells and promotes a pathogenic inflammation. The vicious cycle that is established by the defective CFTR function, recurrent infections and chronic inflammation ultimately results in lung damage and failure (Costantini et al., 2020). Besides bacterial infections by the common CF pathogens *Pseudomonas aeruginosa* and *Staphylococcus aureus*, fungal infections are emerging as relevant medical conditions, with a pivotal role played by the filamentous fungus *A. fumigatus*. Current antifungal drugs target fungi-selective components, such as polyenes, azoles and echinocandins targeting ergosterol or glucan in the fungal cell wall, but present with clinical shortcomings, thus opening up the way for the development of novel drugs. Moved by the hypothesis that differences in the metabolism between fungi and humans may be exploited for therapeutic purposes, we found a potential context-dependent mechanism in CF based on the enzyme sphingosine-1-phoshate lyase (SPL). SPL participates in the sphingolipid metabolism and catalyzes the irreversible degradation of sphingosine-1-phosphate (S1P) to hexadecenal and ethanolamine phosphate. Indeed, CF patients present multiple defects in the sphingolipid metabolism, including reduced levels of S1P in the lung (Veltman et al., 2016), and an inhibitor of SPL could normalize the immune response upon challenge with inflammatory stimuli in a murine model of CF (Veltman et al., 2016). Conversely, sphingolipids are emerging as antifungal targets and increased levels of S1P appear to be toxic to fungi (Kim et al., 2000; Rollin-Pinheiro et al., 2016). Altogether, these pieces of evidence suggest that an increase in S1P levels in both the CF host and the fungus could promote the immune response while impairing fungal virulence. By following this rationale, we first proved by lipidomics that S1P levels were reduced, while those of ceramide increased, in the lungs of CF mice with aspergillosis and that a specific siRNA against murine SPL was able to reduce fungal burden, neutrophil infiltration in the bronchoalveolar lavage and lung histopathology. Second, we proved that increasing S1P levels in the fungus was able to prevent germination of *A. fumigatus* conidia. Since all the piece of the puzzle fitted together, we moved to the identification of inhibitors that could inhibit both the human and the *A. fumigatus* orthologues of SPL in the perspective of developing potential drugs able to perform both anti-inflammatory and anti-microbial activities in CF (Cellini et al., 2023).

All in all, this study demonstrates that the same metabolic pathway in humans and fungi plays different effects depending on the metabolic map in which it operates, as illustrated by the concept of network-based selectivity (Haanstra et al., 2017). It comes that these trans-kingdom metabolic differences may be exploited to develop novel drugs that work at the human-fungal interface by inducing distinct responses that collectively synergize to protect against infection.

## 4 The case of oxalate-degrading pathways

Hyperoxaluria is a condition characterized by increased levels of oxalate, an end-product of metabolism, in the urine. In humans, oxalate comes from two major sources that almost equally contribute to the oxalate endogenous levels in healthy individuals. One source is represented by hepatic metabolism of glyoxylate, that is converted to oxalate by the activity of a lactic dehydrogenase, and the other by the diet with the intake of oxalate-containing food. In the gut, oxalate can be either absorbed and excreted by the kidney, eliminated through the feces upon combining with calcium and forming insoluble calcium oxalate, or degraded by oxalate-consuming bacteria of the microbiota. If oxalate homeostasis is impaired, hyperoxaluria may arise, resulting in the formation of oxalate stones in the kidney and, in the most severe forms, systemically. Hyperoxaluria may derive from genetic deficiencies, namely, primary hyperoxaluria (PHs), due to the accumulation of endogenous glyoxylate. Three forms of PHs have been identified. The most common and severe form is PH1, caused by mutations in the *Agxt* gene encoding L-alanine:glyoxylate aminotransferase (AGT), a liver peroxisomal enzyme specialized in the detoxification of glyoxylate. The other two forms, PH2 and PH3, are less frequent and associated with a milder phenotype, and are caused by mutations in the *Grhpr* and *Hoga1* genes, respectively (Dindo et al., 2019). Hyperoxaluria can also be secondary to other conditions, such as those characterized by fat malabsorption, causing calcium to bind to fatty acids, thus freeing exogenous oxalate to enter the bloodstream, a condition also favored by increased gut permeability (Ermer et al., 2023). The knowledge of the mechanisms regulating oxalate homeostasis have driven the development of innovative therapeutic strategies, such as the FDA-approved Lumasiran (Garrelfs et al., 2021), an siRNA targeting glycolate oxidase, thus reducing the amount of available glyoxylate, the precursor of oxalate, or Nedosiran (Syed, 2023), which targets lactate dehydrogenase, thus preventing the glyoxylate-to-oxalate conversion. In secondary hyperoxaluria, strategies targeting the absorption of exogenous oxalate have been tested. They include the use of oxalate-degrading bacteria, such as *Oxalobacter formigenes* (Daniel et al., 2021), a specialist oxalotroph that uses oxalate as exclusive carbon source. Despite the indisputable potential of *Oxalobacter formigenes* in the reduction of oxalate levels, it appears that functional microbial communities rather than selected species might be more effective at this purpose (Miller et al., 2017). Indeed, the microbiota also contain facultative oxalotrophs that may contribute to the regulation of oxalate levels (Hatch, 2017). All of these studies, however, have focused on the bacterial component of the microbiota while leaving unexplored other potential players, such as the commensal fungi, collectively known as mycobiota. Oxalate-degrading pathways have been classified in two classes by Liu *et al.* (Liu et al., 2021) involving either a direct cleavage of the carbon-carbon bond of oxalate (type I) or the formation of the intermediate oxalyl-CoA, upon which an oxalyl-CoA decarboxylase (OXC) acts to form carbon dioxide and formyl-CoA (type II). In the type I pathway, the cleavage of the carbon-carbon bond of oxalate is performed by either oxalate oxidase or oxalate decarboxylase enzymes, whose expression was found both in fungi and bacteria (Liu et al., 2021). In the type II pathway, the formation of oxalyl-CoA may occur via a formyl-CoA-oxalate CoA-transferase (FRC) or an oxalate-CoA ligase. FRC- and OXC-coding taxa were found to be strictly bacterial (Liu et al., 2021). We began to explore whether the most abundant commensal fungi will express oxalate-degrading pathways. We focused our attention on a type II oxalate-degrading pathway based on the activity of an oxalyl-CoA synthetase, as this enzymatic activity has been reported in *Saccharomyces cerevisiae* (Foster and Nakata, 2014). *Saccharomyces* is abundantly present in human feces although its role as true commensal is disputed (Table 2 and (Hallen-Adams and Suhr, 2017)). Indeed, it likely originates from food and its presence appears to be transient although the potential of modulating the gut microbiota cannot be excluded. We therefore looked for evidence of oxalate-degrading pathways in *Candida* spp. as the mammalian gut represents their primary niche (Hallen-Adams and Suhr, 2017). We could show that *Candida glabrata* (*Nakaseomyces glabrata*) encodes for a protein with a high degree of similarity with the oxalyl-CoA synthetase of *Saccharomyces cerevisiae*. Moreover, superimposition of the two structures revealed conserved residues at the site of oxalate binding and the presence of a P-loop for the binding of ATP required for the reaction (Costantini et al., 2024). In addition, a multiple sequence alignment using the sequences of OXCs from *Escherichia coli*, *O. formigenes*, *Arabidopsis thaliana*, and *S. cerevisiae* suggested that a functional OXC may also be present in *C. glabrata*, supporting the hypothesis that a type II oxalate-degrading pathway may operate in this commensal fungus (Costantini et al., 2024).

**TABLE 2:** Common fungi in gut microbiota studie**s** (adapted from Hallen-Adams and Suhr, 2017).

Genus	Species
*Candida*	*Candida albicans*
	*Candida tropicalis*
	*Candida parapsilosis*
	*Candida glabrata*
	*Candida krusei*
	*Candida lusitaniae*
*Saccharomyces*	*Saccharomyces cerevisiae*
*Penicillium*	*Penicillium aff. commune*
*Aspergillus*	*Aspergillus aff. versicolor*
*Cryptococcus*	
*Malassezia*	*Malassezia globosa*
	*Malassezia restricta*
	*Malassezia pachydermatis*
*Cladosporium*	*Cladosporium aff. herbarum*
*Galactomyces*	*Galactomyces geotrichum*
*Debaryomyces*	*Debaryomyces hansenii*
*Trichosporon*	

All in all, these results suggest that commensal fungi may express oxalate-degrading pathways and contribute to the regulation of oxalate levels in health and disease. It is interesting to note that the trans-kingdom metabolic differences span beyond the human-fungal interface to include bacteria, thus expanding the armamentarium shared by commensal microbes in oxalate homeostasis, and prompting to the development of therapeutic strategies encompassing both bacterial and fungal communities.

## 5 Conclusion

**FIGURE 1:**
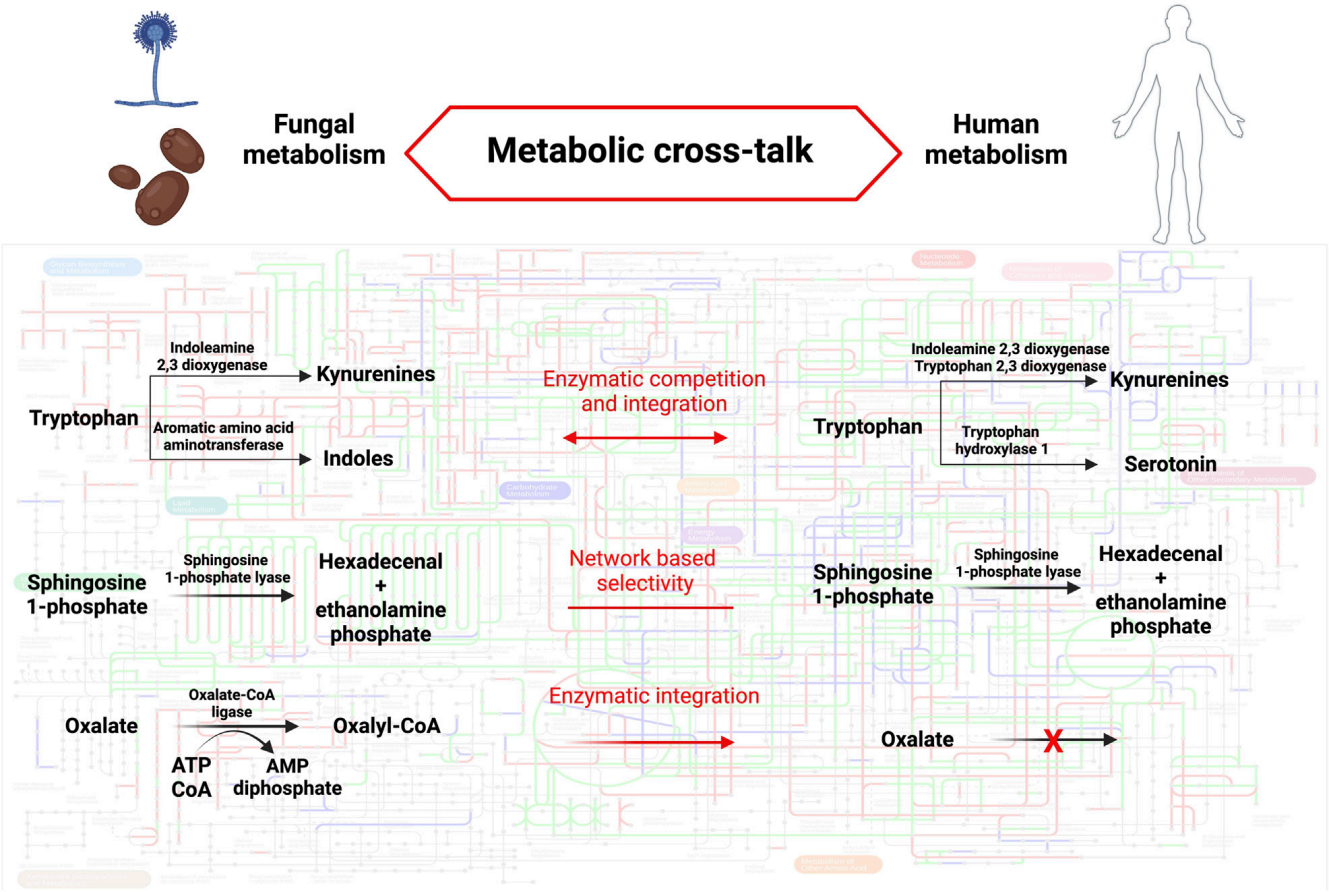
Metabolic cross-talk between humans and fungi. The background metabolic map highlights KEGG orthologous groups present in *Homo sapiens* (red), *Aspergillus fumigatus* (blu) or both species (green). The metabolic pathways described in the text are detailed in fungi (left) and humans (right). The mode of cross-talk is indicated for each pathway (middle). Pathway map was created using iPath3.0 (Darzi et al., 2018). Created with BioRender.com.

Revealing metabolic distinctions between human and fungal enzymatic repertoires could unveil new avenues for therapeutic interventions. As delineated earlier, the outcome of host-fungal interactions can essentially be distilled down to the flow of metabolites within organismal metabolic frameworks and the particular impacts these metabolites exert within each system (see Figure 1). Trp serves as a quintessential example in this regard, acting as a pivotal nexus where shared or distinct enzymatic activities of hosts, bacteria, and fungi converge to regulate the nature and quantity of bioactive metabolites, thereby influencing the outcome of host-fungal interactions. The exploitation of shared or specific enzymes could also modulate host-fungal interactions in various contexts. For instance, inhibiting shared SPL activity in both the host and *A. fumigatus* could prove beneficial in cystic fibrosis (CF), as elevated S1P levels elicit disparate effects in the host and the fungal pathogen, ultimately leading to an enhanced antifungal response. Conversely, employing specific fungal oxalate-degrading enzymes might represent a therapeutic approach in cases of hyperoxaluria. To broaden our understanding of potential metabolic interactions at the host-fungal interface that could be harnessed for therapeutic purposes, it is imperative to deepen our knowledge of fungal metabolism, particularly those species relevant to human health and disease. This necessitates integrating genome analysis with functional biochemistry, with a particular focus on regulatory networks influenced by both external and internal stimuli, in order to adapt metabolic frameworks to the specific context under investigation.

## Data Availability

The original contributions presented in the study are included in the article/Supplementary material, further inquiries can be directed to the corresponding authors.
